# In situ kinetic measurements of α-synuclein aggregation reveal large population of short-lived oligomers

**DOI:** 10.1371/journal.pone.0245548

**Published:** 2021-01-22

**Authors:** Enrico Zurlo, Pravin Kumar, Georg Meisl, Alexander J. Dear, Dipro Mondal, Mireille M. A. E. Claessens, Tuomas P. J. Knowles, Martina Huber

**Affiliations:** 1 Department of Physics, Huygens-Kamerlingh Onnes Laboratory, Leiden University, Leiden, The Netherlands; 2 Centre for Misfolding Diseases, Department of Chemistry, University of Cambridge, Lensfield Road, Cambridge, United Kingdom; 3 Nanobiophysics Group, Department of Science and Technology, University Twente, Enschede, The Netherlands; 4 Cavendish Laboratory, University of Cambridge, Cambridge, United Kingdom; Martin-Luther-Universitat Halle-Wittenberg, GERMANY

## Abstract

Knowledge of the mechanisms of assembly of amyloid proteins into aggregates is of central importance in building an understanding of neurodegenerative disease. Given that oligomeric intermediates formed during the aggregation reaction are believed to be the major toxic species, methods to track such intermediates are clearly needed. Here we present a method, electron paramagnetic resonance (EPR), by which the amount of intermediates can be measured over the course of the aggregation, directly in the reacting solution, without the need for separation. We use this approach to investigate the aggregation of α-synuclein (αS), a synaptic protein implicated in Parkinson’s disease and find a large population of oligomeric species. Our results show that these are primary oligomers, formed directly from monomeric species, rather than oligomers formed by secondary nucleation processes, and that they are short-lived, the majority of them dissociates rather than converts to fibrils. As demonstrated here, EPR offers the means to detect such short-lived intermediate species directly in situ. As it relies only on the change in size of the detected species, it will be applicable to a wide range of self-assembling systems, making accessible the kinetics of intermediates and thus allowing the determination of their rates of formation and conversion, key processes in the self-assembly reaction.

## Introduction

The function of the α-synuclein protein (αS) is associated with its ability to bind to the membranes [1–3] of intracellular vesicles and thought to involve membrane remodeling and vesicle trafficking [4–6]. It mainly localizes at the synaptic terminus where it plays a role in synaptic transmission. The binding of αS to membranes may directly contribute to membrane remodeling by generating curvature [7–9] or, indirectly, by acting as a non-conventional chaperone for the SNARE protein synaptobrevin [10]. Additionally the ability of αS to connect vesicles plays a role in vesicle trafficking at the synapse by controlling the distal synaptic vesicle pool [11]. While the exact functions of the αS protein only now start to become clear, its association with Parkinson’s disease is well documented.

The in vitro aggregation of αS revealed that besides the cross-β fibrils, which represent the end-point of the amyloid aggregation, oligomers are also formed [12–18]. Several works have shown that these oligomeric species are toxic to cells [19, 20], however, most studies rely either on fluorescently labelled αS species, use kinetically trapped oligomers, which may not constitute intermediates of the aggregation reaction, or involve biochemically isolated oligomers and indirect detection [21–24].

**Fig 1:**
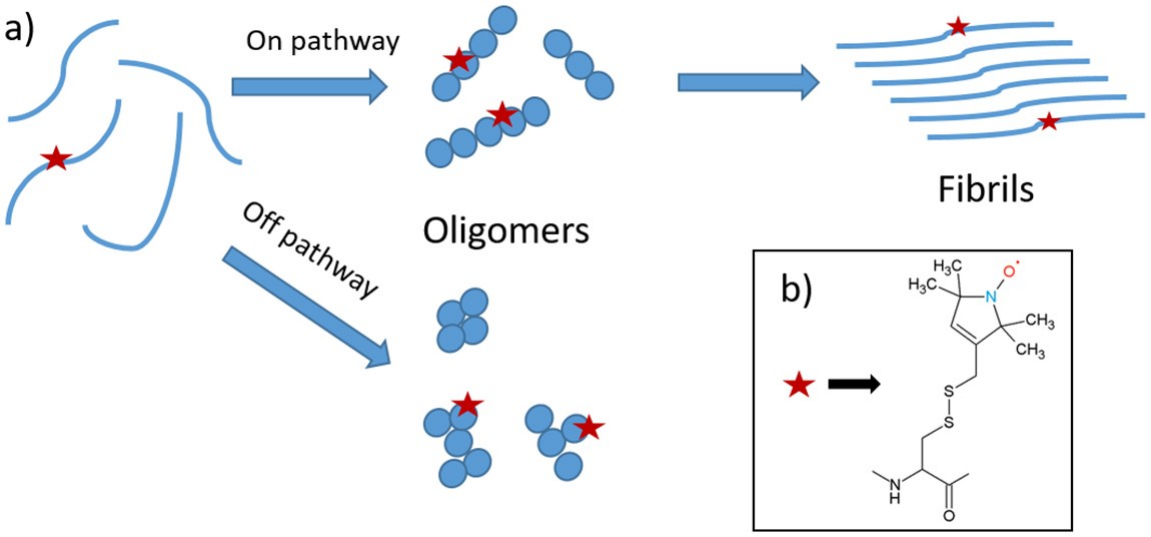
a) Overall reaction pathways and intermediates of α-synuclein aggregation shown schematically. Blue spheres: monomers within oligomers (shapes of oligomers are arbitrary). Red stars: R1 nitroxide spin label (see b). b) Molecular structure of the R1 spin label attached to the protein. No specific kinetic pathways are shown.

While these approaches are invaluable for structural and toxicological studies, they cannot detect the oligomers in situ, to reveal how the oligomers develop in the aggregating solution. Also labelling with the relatively large fluorescent labels and isolation of oligomers may cause a significant modification of the protein or oligomer structure. Thus, there is need for methods to detect such intermediates, directly in solution during the aggregation reaction, without the need for large fluorescence labels. Here we present an approach that closes this gap: In situ continuous wave electron paramagnetic resonance (EPR). This method measures the rotational diffusion time of spin labelled objects in liquid solution at room temperature. Sensitivity of the EPR lineshape of nitroxide-spin labels to time scales in the nano-second regime ensures that the aggregates of interest (see Fig 1) are well covered.

To apply the method, the αS is labelled with the spin label shown in Fig 1b. This label is attached to a cysteine at position 56 that was introduced by site selective mutagenesis, resulting in the construct R1-αS, where R1 stands for the spin label. We chose to place the spin label at position 56, because this is a region of αS expected to be immobilized in the aggregates. Positions in the C-terminus, residues 100–130, and close to the N-terminus, are avoided because they are expected to remain flexible in most aggregation models. The spin label itself, see Fig 1b, is significantly smaller than commonly used fluorescent labels, and the scarceness of unpaired electrons in biological systems makes the method background free.

We use this methodology to reveal the appearance of an intermediate aggregated species. We present a kinetic model that explains the development of intermediates and fibrils.

## Material and methods

### Protein expression and labeling

Mutagenesis, protein expression and purification were performed as described previously [25, 26]. The mutated protein was spin labeled following a standard protocol. The αS56 (cysteine mutant in position 56) was reduced with a six-fold molar excess per cysteine with DTT (1,4-dithio-D-threitol) for 30 minutes at room temperature. Removal of DTT was done by passing the samples twice through a Pierce Zeba 5 ml desalting columns. Immediately afterwards, a ten-fold molar excess of the MTSL spin label [(1-oxyl-2,2,5,5-tetramethylpyrroline-3-methyl)-methanethiosulfonate] was added (from a 25 mM stock solution in DMSO) and incubated for one hour in the dark at room temperature. The initial protein concentration was 250 μM, the spin label concentration was 2.5 mM. After this, free spin label was removed by two additional desalting steps. Protein samples were applied onto Microcon YM-100 spin columns to remove any precipitated and/or oligomerised proteins and then diluted in buffer (10 mM Tris-HCl, pH 7.4). Due to the high reactivity of the MTSL and the easily accessible cysteine, near stochiometric labeling was achieved under these conditions [27]. To determine the labelling degree, protein concentration was measured via the extinction at 280 nm (ε = 5600 cm^-1^ M^-1^), and the spin concentration via the double integral method of EPR described below. Samples were stored at -80°C.

### Sample preparation

#### Description of experiment

A stock solution of spin-labeled α-synuclein (concentration between 150 μM and 250 μM) was diluted into 3 mL of buffer (10 mM Tris-HCl, pH 7.4), to a final concentration of 10 μM. The solution also contained 90 μM wild-type α-synuclein for diamagnetic dilution (see below). The solution was divided into three 2 ml LoBind Eppendorf tubes, resulting in a volume of 1 ml per Eppendorf tube. The experiments were carried out over five days. After an initial measurement was taken at the time the spin-labelled protein was diluted (t = 0), the samples were allowed to aggregate on a thermomixer (Eppendorf, Thermomixer comfort) with a speed of 1000 rpm at 37°C. At each time point, 40 μL samples were drawn from the aggregation solution and kept in the fridge at 4°C. From these, samples for EPR and Thioflavin T (ThT) fluorescence measurements were made. At the beginning samples were collected every 3 hours, at later times the intervals were longer (see text).

The data shown are from the first of these Eppendorf tubes, and the data set is referred to as LV1, low aggregation volume number 1. Data from additional experiments are shown in the SI of S1 File only: One data set obtained using the solution from the second Eppendorf (LV2), which has similar behavior to LV1 (see S4b Fig of S1 File). A second set of aggregation experiments was performed at a lower surface to volume ratio: These data sets are referred to as HV1 and HV2, and their aggregation conditions and results are described in the SI of S1 File, the data are shown in S4c and S4d Fig of S1 File.

### EPR measurement conditions

The 9 GHz, continuous-wave EPR spectra were recorded using an ELEXSYS E680 spectrometer (Bruker, Rheinstetten, Germany). The measurements were done under the following conditions: room temperature, a microwave power of 0.63 mW and a modulation amplitude of 0.25 mT at a modulation frequency of 100 kHz. The time expended on each measurement was adapted according to the spectral lineshape, i.e., the aggregation time, and they could last from 3 to 8 hours. At long aggregation times the spectral amplitude decreases due to line broadening, and therefore, to obtain the desired signal-to-noise ratio, a longer accumulation time is needed. In practice, we inspected the signal-to-noise ratio of each EPR spectrum after a given accumulation time and increased the measurement time if the spectral quality was not yet sufficient. Glass micropipettes of a volume of 50 μL (Blaubrand Intramark, Wertheim, Germany) were filled with 20 μL of the sample for each measurement. The spin concentration was determined by comparing the double integral of the EPR spectra with the double integral of a reference sample (MTSL, 100 μM). The spin concentrations were ≈ 10 μM for a total protein concentration of 100 μM.

### Simulations of EPR spectra

MATLAB (version 9.4.0.813654, R2018a, The MathWorks, Inc., Natick, MA, USA) and the EasySpin package (5.2.4) were used for simulations of the EPR spectra [28]. The parameters of the simulation were manually adjusted to agree best with the experimental spectra. For all simulations, an isotropic rotation of the nitroxide (S = 1/2) was utilized. The following g-tensor values were used: g = [2.00906 2.00687 2.00300]. These values were obtained in previous experiments [29] and we used these values for the simulations presented here. The spectra were simulated with a superposition of four components: two fast fractions using the “Garlic” function and a medium and a slow fraction using the “Chili” function. The principal values of the ^14^*N* hyperfine coupling tensor were A_xx_ = A_yy_ = 13 MHz and A_zz_ = 110 MHz. For the slow component A_zz_ = 106 MHz was used instead. A Gaussian component with a linewidth of 0.12 mT was used for all simulations. The spectrum obtained at t = 0 could be simulated with a single component, the fast fraction. The τ_r_ obtained at t = 0 was kept constant for all other simulations. From t = 3 hours a new component appeared, τ_r_ = 0.24 ns which we attribute to free spin label. Its contribution to the spectra never exceeded 10%. Optimal τ_r_ values of the medium and slow components were derived from later time-point spectra and then kept constant for the entire series. For each time point, the relative contribution of the four components was optimized considering all time points.

Because of the diamagnetic dilution, which increases the spin-spin distance, the only spectral changes expected derive from mobility differences: By diluting with wt-αS the distances between spin labels should exceed those to which cw-EPR could be sensitive, also in aggregates. Exchange interaction could be visible at around 0.5 nm and below, and up to 1 nm under specific circumstances, dipolar interactions, neither of which are likely to occur with a 10 fold diamagnetic dilution.

### Fitting of kinetics

The EPR measurements yield, after processing, the monomer-equivalent concentrations of monomers, intermediates and fibrils at different time points. We fit a minimal model of the aggregation of αS into fibrils that additionally includes the formation of oligomers directly from monomers with rate constant *k*_*o*_, for details on the meaning of *n*, see below. Oligomer dissociation proceeds at rate constant *k*_*d*_. Oligomers can be converted to fibrils by rate constant *k*_*c*_. Fibrils grow by addition of monomers to their ends with rate constants *k*_*+*_ and we allow for the presence of secondary processes, i.e. the possibility of fibrils to multiply in a monomer-independent manner, e.g. by fragmentation, by rate constant *k*_*2*_. We do not include oligomeric species formed by a secondary process. More detailed descriptions of these kinetic models can be found for example in Meisl et al [30–32].

Invoking conservation of mass, *m*_*tot*_ *= m(t) +M(t) +O(t)*, the aggregation process can be described by the following set of differential equations:

$$\frac{dP(t)}{dt}=k_{c}\;O(t)+k_{2}(m_{tot}-n\;O(t)-m(t))$$

$$\frac{dM(t)}{dt}=2k_{+}P(t)m(t)$$

$$\frac{dm(t)}{dt}=-2\;k_{+}P(t)m(t)-nk_{o}m{\left(t\right)}^{n}+n\;k_{d}O(t)$$

$$\frac{dO(t)}{dt}=k_{o}m{\left(t\right)}^{n}-(k_{c}+k_{d})O(t)$$

Where *M(t)* is the monomer-equivalent concentration of fibrils, *P(t)* and *O(t)* are the number concentrations of fibrils and of oligomers, respectively, *m(t)* is the free monomer concentration, and *n* the oligomer reaction order, which, under specific conditions (see Discussion), can be related to oligomer size. These equations were numerically integrated and fitted to the data by least squares minimization. This model assumes that oligomers are on-pathway, but as we show in the SI of S1 File, the data are also consistent with an off-pathway model. We assumed a monomer-concentration independent secondary mechanism, such as fragmentation, based on previous studies of αS [32, 33] aggregation into amyloid fibrils and the fact that vigorous shaking tends to induce fragmentation [34]. Additionally, given that the experiments were only recorded at a single initial monomer concentration, they do not provide strong constraints on the reaction orders of both oligomerisation, *n*, and the secondary process [35]. The data provide constraints for a number of parameters: oligomers are in fast equilibrium with monomers, thus the equilibrium constant, *k*_*o*_*/k*_*d*_ can be determined accurately but only an approximate lower bound can be given for the individual rates (corresponding to the requirement that the oligomerization reaction proceeds fast enough to be in effective equilibrium relative to monomer depletion). This lower bound is shown in the fits, see Results. Equally, the rates of elongation, the secondary process and conversion are interdependent (as is the case in all unseeded aggregation reactions) and thus only the products *k*_*+*_*k*_*c*_ and *k*_*+*_*k*_*2*_ can be constrained. Finally, the data show only a weak dependence on the reaction order of the oligomerization reaction, *n*.

## Results

**Fig 2:**
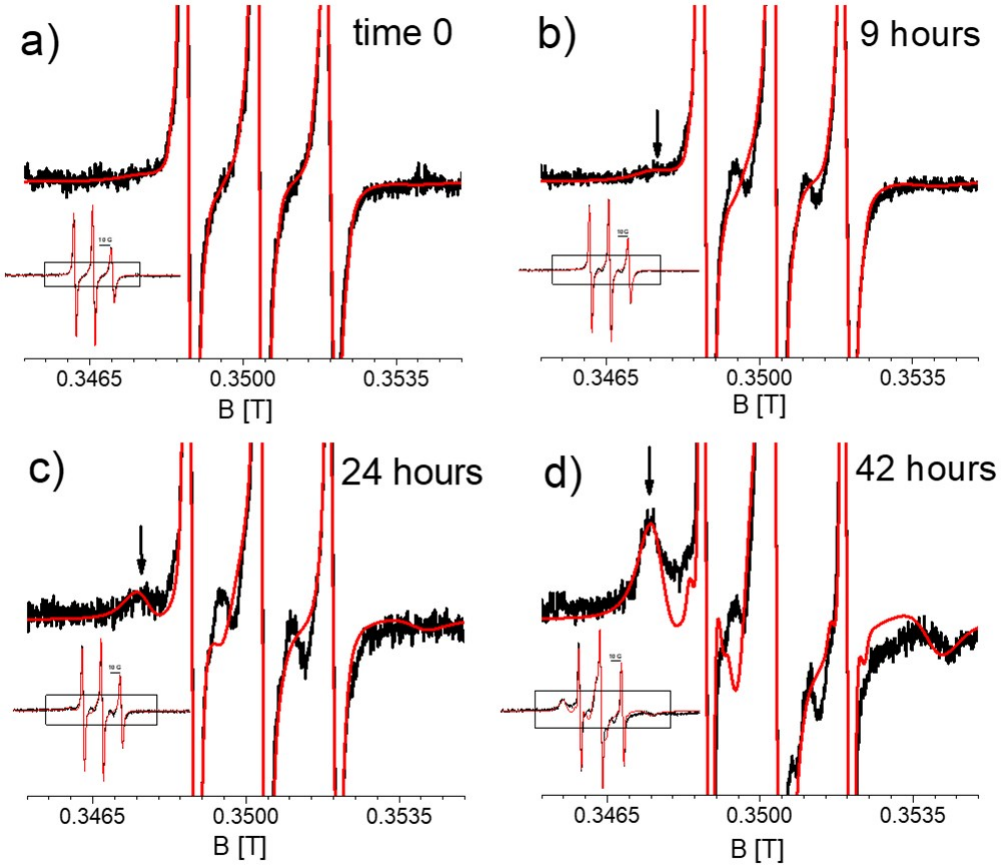
Room temperature EPR spectra of α-synuclein (R1-αS) at different time points of aggregation. Full spectra: Inset, the box shows the region zoomed into. Zoomed-in spectra: Amplitude expanded four-fold with respect to inset. a) Start of aggregation (t = 0). b) 9 hours of aggregation. c) 24 hours of aggregation. d) 42 hours of aggregation. Black: Experimental spectra. Red: Simulated spectra. Arrow: feature of broad spectral component (see text).

The aggregation of 100μM α-synuclein was monitored at pH 7.4 and 37°C under rapid shaking (1000rpm), over the course of 120 hours. Fig 2 shows the development of the EPR spectra of R1-αS over time for four selected time points, the full set of spectra is shown in S1 Fig of S1 File. The spectra at the start of the aggregation and after nine hours of aggregation (9 h) are dominated by the three narrow lines typical of nitroxides in fast rotational motion.

Starting at nine hours a new component with a broader linewidth (marked by an arrow) develops, that increases in amplitude with time. Its lineshape is due to a nitroxide with a slower rotation and shows that a fraction of R1-αS becomes more immobilized as the aggregation progresses. Line broadening by spin-spin interaction can be excluded, because the R1-αS was diluted in a 1: 9 ratio with wt-αS, which increases the distance between the spins of the nitroxides sufficiently to suppress spectral effects of their interaction (diamagnetic dilution, see Materials and methods).

**Fig 3:**
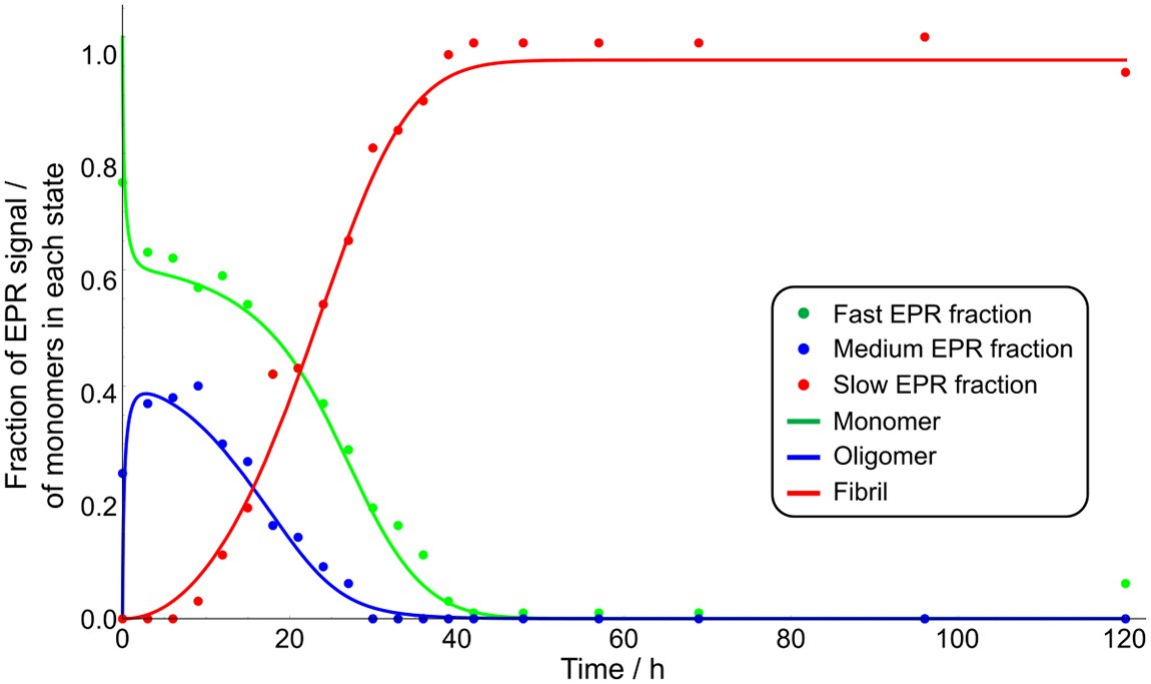
Aggregation of α-synuclein as a function of time derived from EPR. Amount of fast fraction (green dots) caused by monomers. Amount of medium fraction (blue dots) assigned to oligomers. Amount of slow fraction (red dots) assigned to fibrils. The solid lines are the fractions of monomer, oligomer and fibrils predicted from the best fit of the model, with a reaction order of n = 7.

By spectral simulation [28], three components can be extracted, referred to as the fast, medium and slow components. The slow and fast components are adjusted from the spectra at the end and the beginning of the aggregation, respectively. From these building blocks the intermediate-time spectra are constructed, revealing the medium component and enabling the recognition of the slow component marked by the arrow in Fig 2b and 2c, for example. The respective rotation correlation times (τ_r_) of the three components are given in Table 1, and their lineshape is shown in S2 Fig of S1 File. The τ_r_ value of the fast fraction agrees with the τ_r_ values of monomeric αS with the spin label at the position of R1- αS [36]. The amount by which each fraction contributes to the spectra is shown in Fig 3.

**Table 1:** Rotation correlation time (τ_r_) of R1-αS in the three fractions observed by EPR.

	fast	medium	slow
**τ**_**r**_ **(ns)**	0.40	4.00	10

The magnitude of τ_r_ relates to the size of the aggregated species, where, qualitatively, a longer τ_r_ corresponds to a larger species, see for example M. Hashemi Shabestari et al [37]. Therefore, the medium fraction can be attributed to smaller aggregates than the slow fraction.

The fast component decays to zero within the first 40 hours. The medium fraction is already present at the earliest time point (25%) and increases quickly to a maximum value of 40%. At 30 hours, this component has fully decayed. The medium fraction thus disappears before the monomer fraction is fully depleted. The slow fraction appears after 10 hours, it increases to reach 100% at 40 hours, and then remains at its plateau level until the end of the measurements, at 120 hours.

**Fig 4:**
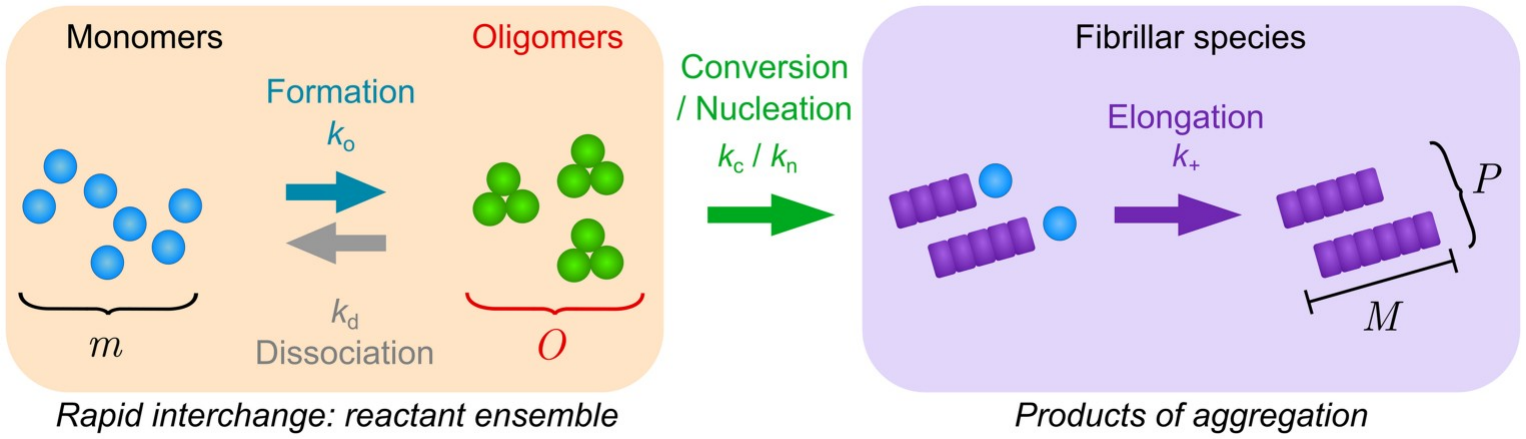
Schematic of the kinetic model used to fit the experimental data, showing species, rate constants and processes considered in the kinetic model. Because monomers and oligomers reach pre-equilibrium on a timescale significantly faster than that of the measurement intervals, altering this model to make oligomers off-pathway does not affect the fit quality. As such, the oligomers cannot be resolved as on- or off-pathway and must instead be considered part of the reactant ensemble at this experimental time resolution [42]. Left: Oligomer formation (*k*_*o*_) and dissociation (k_d_) interconvert monomers (m) and oligomers (O). Conversion (*k*_*c*_) or nucleation (*k*_*n*_), green arrow, leads to fibrils (right). Elongation, (*k*_*+*_) grows existing fibrils, M denotes the monomer equivalent fibril concentration and P the number concentration of fibrils.

The kinetics of monomer, fibrillar and oligomeric fractions determined in this way were used in a kinetic analysis to determine the mechanism of aggregation and the respective reaction rates. The minimal model that was able to describe the data included an oligomeric species, formed directly from monomer, that can convert to growth-competent fibrils in a unimolecular reaction. A model in which these oligomers cannot convert to fibrils, and fibril nucleation instead proceeds by a separate process is also consistent with the data (see SI of S1 File). Fibrils grow by addition of monomers and existing fibrils can lead to the formation of new fibrils via a secondary process, but they do not significantly affect the production of oligomers. The processes and rate constants considered in this model are shown in Fig 4.

## Discussion

Methods to determine the time course of amyloid aggregation are important as intermediates of aggregation are believed to be key toxic species [19, 20, 38–40]. Here we focus on αS, an amyloid protein related to Parkinson’s disease, whose physiological role is yet to be determined.

Under the conditions investigated here, αS is expected to fibrilize slowly, i.e. over the course of days. We track the aggregation of α-synuclein in situ by EPR lineshape changes, which reflect the mobility of the spin label in R1-αS.

Following the time course of the process over the period of 5 days, three distinct fractions are observed: A fast fraction is due to monomeric R1-αS. The fast fraction decays with time and at 40 hours has disappeared. The second fraction, with medium mobility, grows with time with a maximum early in the aggregation reaction followed by a decay. The transientness of this species suggests that this fraction represents oligomers which are higher in energy than the fibrillar end product of the aggregation reaction. The slow fraction appears after a lag time and then grows to a plateau value that is close to the full population. To investigate the influence of our chosen experimental setup, in particular the availability of surfaces, which is known to play an important role in controlling the aggregation kinetics, we also investigated the reaction under the same conditions, but at increased volumes. As expected, decreased rates are observed when the surface to volume ratio is decreased, but the conclusions drawn remain robust (details see SI of S1 File).

### Relation of the EPR derived fractions to the aggregation state of αS

The contribution to the overall EPR signal reflects the relative number of αS proteins in this particular aggregation state. Thus, the fraction with a fast rotational correlation time corresponds to the fraction of total αS that is in the monomeric state. In addition to the monomeric species, two types of aggregates can be distinguished by EPR, those with a slow rotational correlation time, corresponding to large aggregates and those with a medium rotational correlation time, corresponding to intermediate aggregate sizes. The time dependence (Fig 3) shows that intermediate-sized aggregates are formed initially, their time course closely resembling that of the monomer after the initial measurement. The larger aggregates are only formed at a later stage.

It is not possible to derive the exact size of the aggregate, because of the local mobility of the spin label, i.e. the rotation about the single bonds that link the pyrolidine ring to the protein backbone (Fig 1b), see M. Hashemi Shabestari et al [37] for a more detailed discussion. In the present context, a lower limit of the size of the aggregate can be estimated from the ratio of the τ_r_ values of the aggregates with respect to the monomer, showing that the medium fraction comprises minimally ten monomers, the slow fraction at least 25 monomers. We have discussed the factors entering such estimates in detail in M. Hashemi Shabestari et al [37].

The exponential increase of ThT activity appears around 35 hours (S3 Fig of S1 File), consistent with the medium fraction consisting of non-fibrillar aggregates. The slight time shift between the appearance in time of the large aggregate fraction as measured by EPR and the ThT fluorescence (see SI of S1 File) (S3 Fig of S1 File) is likely due to the poor sensitivity of ThT for structures with less beta-sheet content [41]. Thus, we will refer to the medium fraction as oligomeric and the slow fraction as fibrillar.

Based on these observations we propose the following reaction scheme: Oligomers of size *n* form directly from monomers with rate constant *k*_*o*_ and dissociate with rate constant *k*_*d*_. They can be converted to fibrils by rate constant *k*_*c*_. Fibrils in turn grow by addition of monomers to their ends with rate constants *k*_*+*_ and we allow for the presence of a monomer-concentration independent secondary process, such as fibril fragmentation, by rate constant *k*_*2*_. Based on the EPR data, the oligomeric species appear to be in fast equilibrium with monomers on the timescale of the aggregation reaction. Indeed, we find that the rates of oligomer formation and dissociation are fast with *k*_*d*_ > 0.2 h^-1^ and only their equilibrium ratio being well constrained. When the available surface area is decreased, the dissociation rate decreases somewhat, although the timescale of dissociation remains comparable to that of monomer depletion due to aggregate formation (see S1 File). Therefore, monomeric and oligomeric species should be considered part of the same ensemble of reactants for the purposes of a kinetic description. In other words, whether new fibrils are formed directly from monomers or by conversion of oligomeric intermediates is thus not distinguishable based on these kinetic measurements alone [42]. We verify this observation also by showing that fits to a model where oligomers cannot convert to fibrils, and fibrils are instead formed directly from monomer, describe the data equally well (see S5 Fig of S1 File). All the data are best fit by an oligomer reaction order of *n* = 7 (see Fig 3), but the dependence of the goodness of fits on this reaction order is weak, and reasonable fits can be achieved also with smaller or larger reaction orders, such as *n* = 10, the lower limit predicted for the oligomer size based on our EPR data alone (see S6 Fig of S1 File). It is worth noting that a kinetic analysis as presented here yields reaction orders, rather than oligomer sizes directly. In the simplest interpretation, i.e. when the reaction modelled is a single elementary step, the reaction order is indeed equivalent to the oligomer size. However, in more complex reactions, e.g. when heterogeneous nucleation on a surface is involved, reaction orders can be significantly smaller than the oligomer size. The observation that reduction of the surface to volume ratio results in slower oligomer formation, dissociation and conversion is consistent with surfaces playing a key role in these processes. Assuming *n* = 7, the best fit yields *k*_*d*_ > 0.2 h^-1^ and *k*_*o*_*/k*_*d*_ = 2.0x10^24^ M^-6^, *k*_*+*_*k*_*2*_ = 70 M^-1^h^-2^ and *k*_*+*_*k*_*c*_ = 270 M^-1^h^-2^. Note that only the combined rates of nucleation and elongation can be constrained; this is a result of the fact that measurements of the mass concentration of aggregates in the absence of seeds are determined only by a product of these rates, rather than the individual rates [35]. Typical elongation rates are on the order of 10^6^ M^-1^h^-1^ [43], thus conversion, *k*_*c*_, is likely to be orders of magnitude slower than dissociation, *k*_*d*_. A key result is that the oligomers are formed directly from monomer, also in the absence of fibrils, and thus constitute primary oligomers, rather than secondary oligomers, i.e. they are potential intermediates of primary nucleation, not of secondary nucleation, as observed for example in the aggregation of Aβ42, one of the main proteins that aggregate in Alzheimer’s disease [44]. Furthermore, the fast oligomer dissociation rate compared to rate of conversion of oligomers into fibrils indicates that most oligomers dissociate before they can convert into fibrils. In Cremades et al [19] two types of oligomers were identified, type A and type B. Type A oligomers are smaller than and dissociate more readily than type B ones, and both are intermediates of fibril formation; in Dear et al [45] it was shown that these oligomers too predominantly dissociate rather than convert into fibrils. Thus, while the dynamics of the oligomer fraction observed here more closely resembles that of type A than type B oligomers of Cremades et al [19], the significant oligomeric fraction we observe here suggests that the majority of oligomers we detect are not present in single molecule experiments. While the different label may play a role, the fact that our technique requires neither dilution nor separation is likely to be the main source of the observed differences. Given our finding of a fast dissociation rate, we would expect the oligomeric species we detect here in-situ to dissociate significantly upon the dilution that is required to obtain single molecule data. The in-situ measurement by EPR allows us to detect these meta-stable species, which are too short-lived to be measured by other techniques.

## Conclusion

We have demonstrated the power of EPR to measure the presence of oligomeric species over the time course of the aggregation reaction, without need for dilution, size exclusion or other techniques that could potentially alter the size distribution. We find that a large population of non-ThT active intermediates form during the aggregation of αS at pH 7.4 and 37°C. These oligomeric species are in fast exchange with the monomer pool and thus the data are consistent with them being intermediates on the primary nucleation pathway. The data are not consistent with the detected species being secondary oligomers, i.e. formed via a process that is catalyzed by existing fibrils, a mechanism that is believed to be the main source of oligomers during the aggregation of Aβ42. Oligomers are short lived on the timescale of the aggregation reaction and most disappear by dissociation, not by conversion to fibrils. Additional studies with different spin label position in αS could provide local information about the aggregates, if they display different degrees of immobilization, I.e. different τ_r_ values. We envision that our combination of non-disruptive oligomer detection and kinetic analysis will be applicable to study the effect of a range of conditions on the oligomer formation reaction of αS and other amyloid forming proteins.

## Supporting information

S1 File (PDF)
