# Supplementary material for: In situ kinetic measurements of α-synuclein aggregation reveal large population of short-lived oligomers
Source: PLoS One. 2021 Jan 22;16(1):e0245548. doi: 10.1371/journal.pone.0245548 (PMC7822277; doi:10.1371/journal.pone.0245548)
Supplement: S1 File — (PDF) [file pone.0245548.s001.pdf]

Supporting information

ThioflavinT fluorescence

The samples were monitored by the standard Thioflavin T (ThioT) fluorescence assay[1]. In summary, 5 µL of sample solution were dissolved into 2 mL of a solution 5 µM of ThioT and mixed for 30 seconds. The sample was excited at 457 nm and the fluorescence was observed in the range of 475 nm to 600 nm (Varian Cary Eclipse, San Jose, CA, USA). The fluorescence increase was measured with respect to the ThioT blank without the peptide.

Comparison of kinetics using different reaction orders

Because the data are recorded at a single monomer concentration, we cannot obtain strong constraints on the reaction orders. To showcase this fact, Fig. S6 shows the fits with the a reaction order n=7, which was used in the main text, as well as a smaller reaction order n=3.

Comparison of kinetics for on-path versus off-path model

To verify that the data are consistent with the oligomers being off-path as well as with the oligomers being on-path, we show here the fits of an off-path model.

The off-path model is given by

$$\frac{dP(t)}{dt} = k_n\, m(t) + k_2\big(\mathrm{m_{tot}} - n\, O(\mathrm{t}) - m(t)\big)$$

$$\frac{dM(t)}{dt} = 2k_+P(t)m(t)$$

$$\frac{dm(t)}{dt} = -2\, k_+P(t)m(t) - nk_o m(t)^n + n\, k_d O(t)$$

$$\frac{dO(t)}{dt} = k_o m(t)^n - k_d\, O(t)$$

Where the parameters are as defined in the off-path model, but now there is no conversion of oligomers to fibrils (k_c=0) and we have defined a primary nucleation rate, k_n, that accounts for formation of fibrils directly from monomer. We have not explicitly included a reaction order for this process because it cannot be constrained based on the current data at a single monomer concentration.

Additional datasets for αS aggregation and analysis

To test the reproducibility of the aggregation and the robustness of our conclusions additional aggregation experiments were performed. One dataset under the same conditions as described in the main text, adding the data set LV2 to the set described in the main text, and two more datasets, HV1 and HV2, to investigate the importance of surfaces and the sample volume on the effect of shaking. All datasets and their fits are shown in Fig. S4.

Samples for HV1 and HV2 have the same composition as LV1 and LV2, but the aggregation volume was 1.5 ml in a 2.0 ml LoBind Eppedorf tube, effectively decreasing the surface to volume ratio and most likely also the effectiveness of shaking compared to the LV samples, which contained only 1.0 ml of solution (see main text).The dataset of LV2, see Fig. S4 closely resembles that of LV1, showing the reproducibility of the experiments.

For the HV samples, we find that the rates of all processes are decreased when the surface to volume ratio is decreased. This finding can be rationalized by the fact that the oligomer associated process, namely oligomer formation, dissociation and conversion are likely to be catalyzed by interactions with surfaces and that the shear forces, which induce fragmentation, are likely to be stronger when the Eppendorfs are not completely filled. Specifically, we find that the oligomer conversion rate is most significantly affected, whereas the effect on the oligomer formation and dissociation rates, as well as the secondary rate is less pronounced. Notably, the equilibrium constant of oligomer formation remains essentially unchanged, consistent with the introduction of a catalyst, such as a surface that aids oligomer assembly, while leaving the stability of the products unchanged. These data are still consistent with our conclusions that oligomers mainly dissociate rather than convert, that the data are consistent with oligomers being either on- or off-path to fibril formation, and that the oligomers are primary oligomers, formed directly from monomer.

The detailed rates are given in table S1.

Table S1: The rate constants obtained from fits of the different datasets to an on-path model with a reaction order *n*=7. Here k_+ is the elongation rate, in units of $M^{-1}h^{-1}$, k_2 is the rate of the secondary process in h^{-1}, k_o is the oligomer formation rate in units of $M^{-6}h^{-1}$, k_d is the oligomer dissociation rate in units of h^{-1}, and $K_{equ}=k_o/k_d$ is the equilibrium constant of oligomers, in units of M^{-6}. Sample conditions given in the text. In short: LV samples: aggregation volume 1.0 ml, HV: 1.5 ml, all in 2 ml LoBind Eppendorf tubes

samples	k_+*k_2	k_+*k_c	k_o	k_d	K_{equ}
LV1*	69.86	265.86	4.46E+23	0.22	1.99E+24
LV2	21.37	197.82	2.85E+25	21.91	1.30E+24
HV1	23.87	3.66	1.01E+22	0.01	8.49E+23
HV2	17.29	8.33	2.75E+22	0.030	9.09E+23

*also given in main text

Supplementary figures

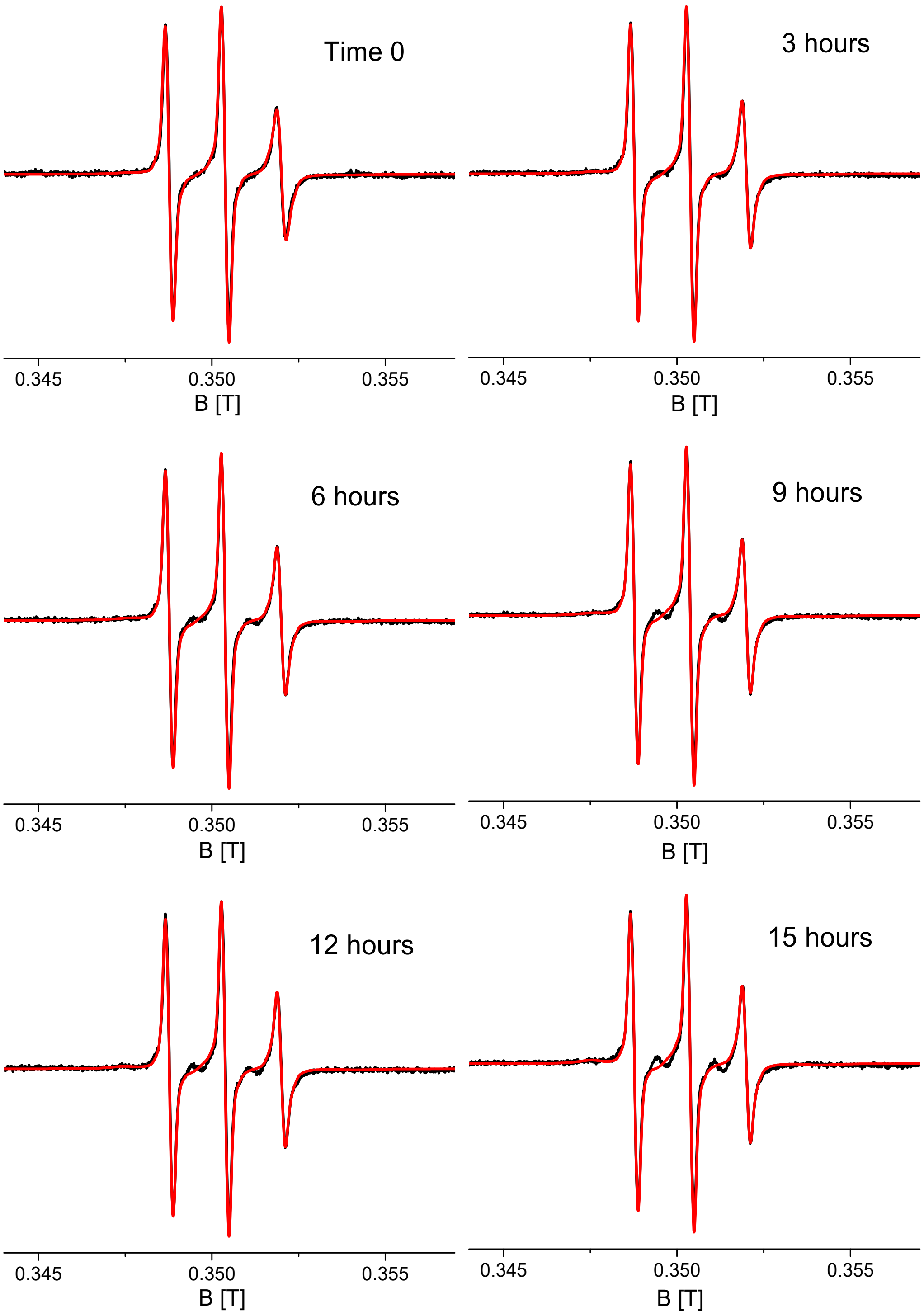

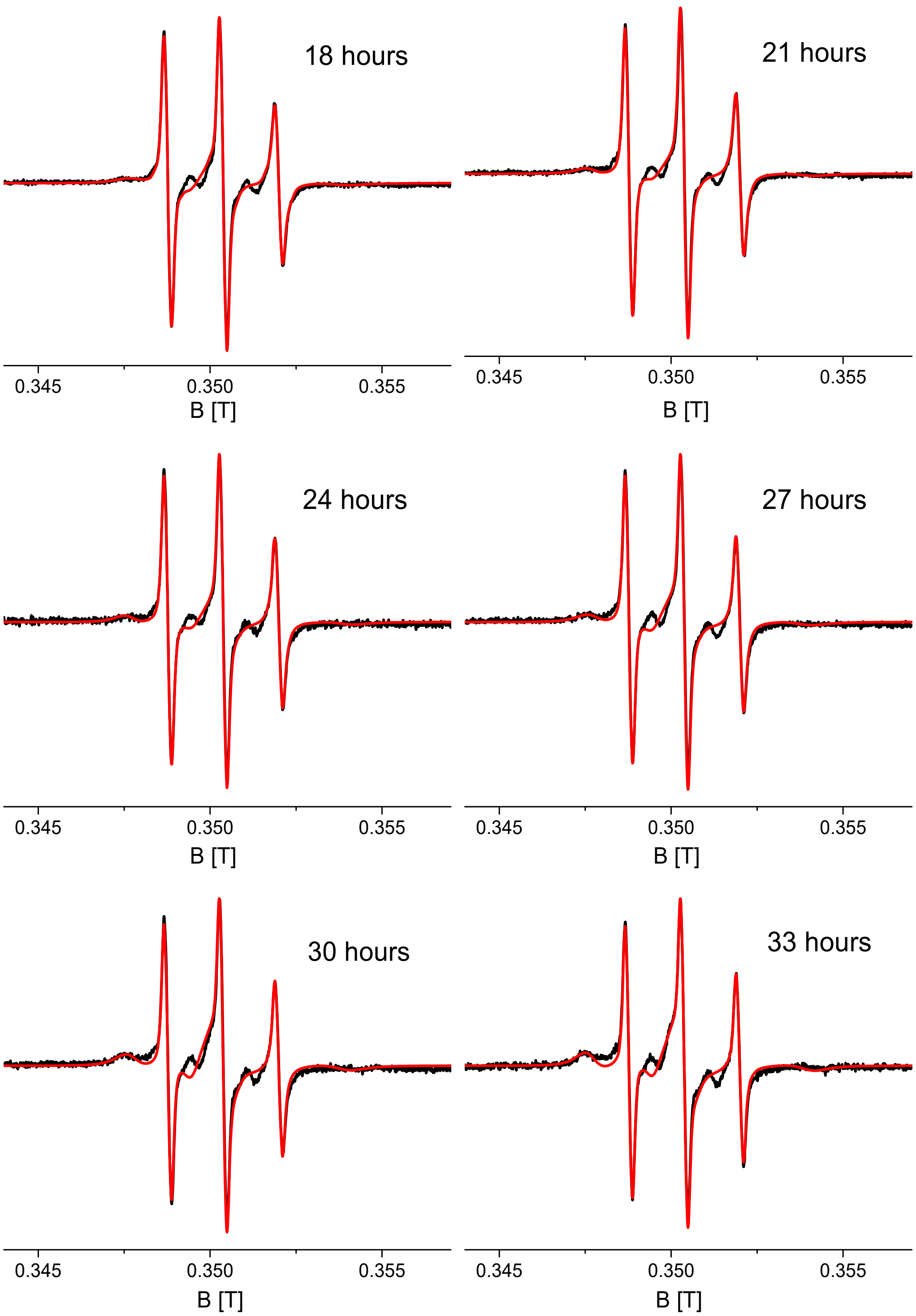
18 hours
21 hours
24 hours
27 hours
30 hours
33 hours
0.345
0.350
0.355
B [T]

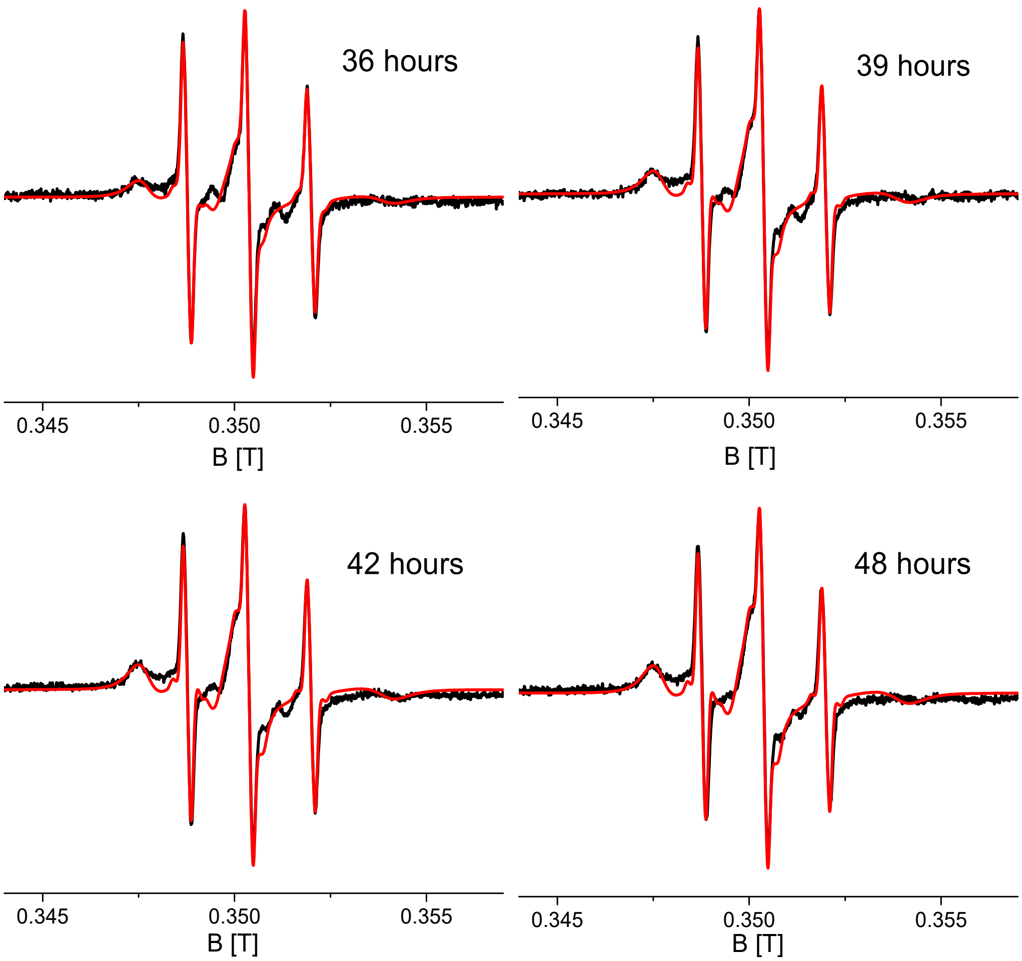
36 hours
0.345
0.350
0.355
B [T]
39 hours
0.345
0.350
0.355
B [T]
42 hours
0.345
0.350
0.355
B [T]
48 hours
0.345
0.350
0.355
B [T]

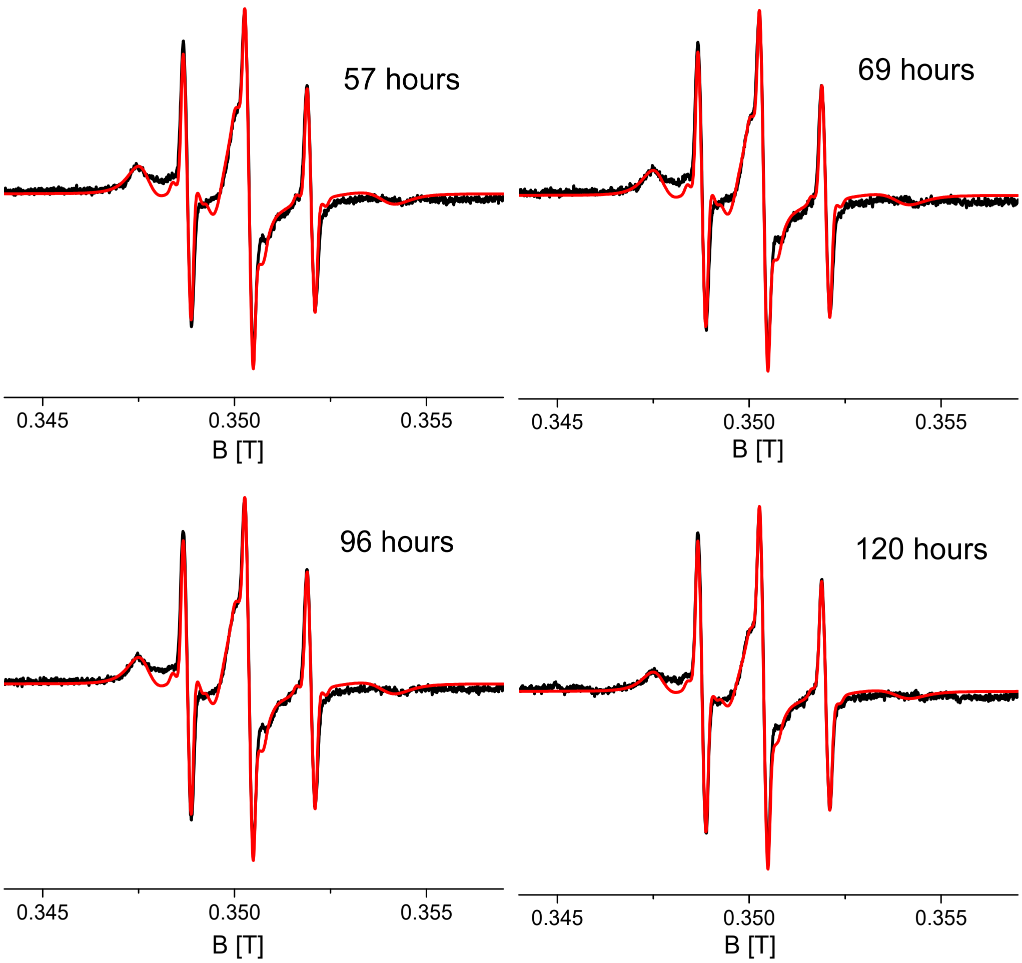

Figure S1. Room temperature 9 GHz EPR spectra of R1- αS(56) for the entire time point series taken during aggregation. Black: Experimental spectra. Red: Simulated spectra. Remaining experimental conditions and detailed description: See main text. Additional lines, see 15 h spectrum at 0.34955 T and at 0.3525 T attributed to biradical formation by free spin label.

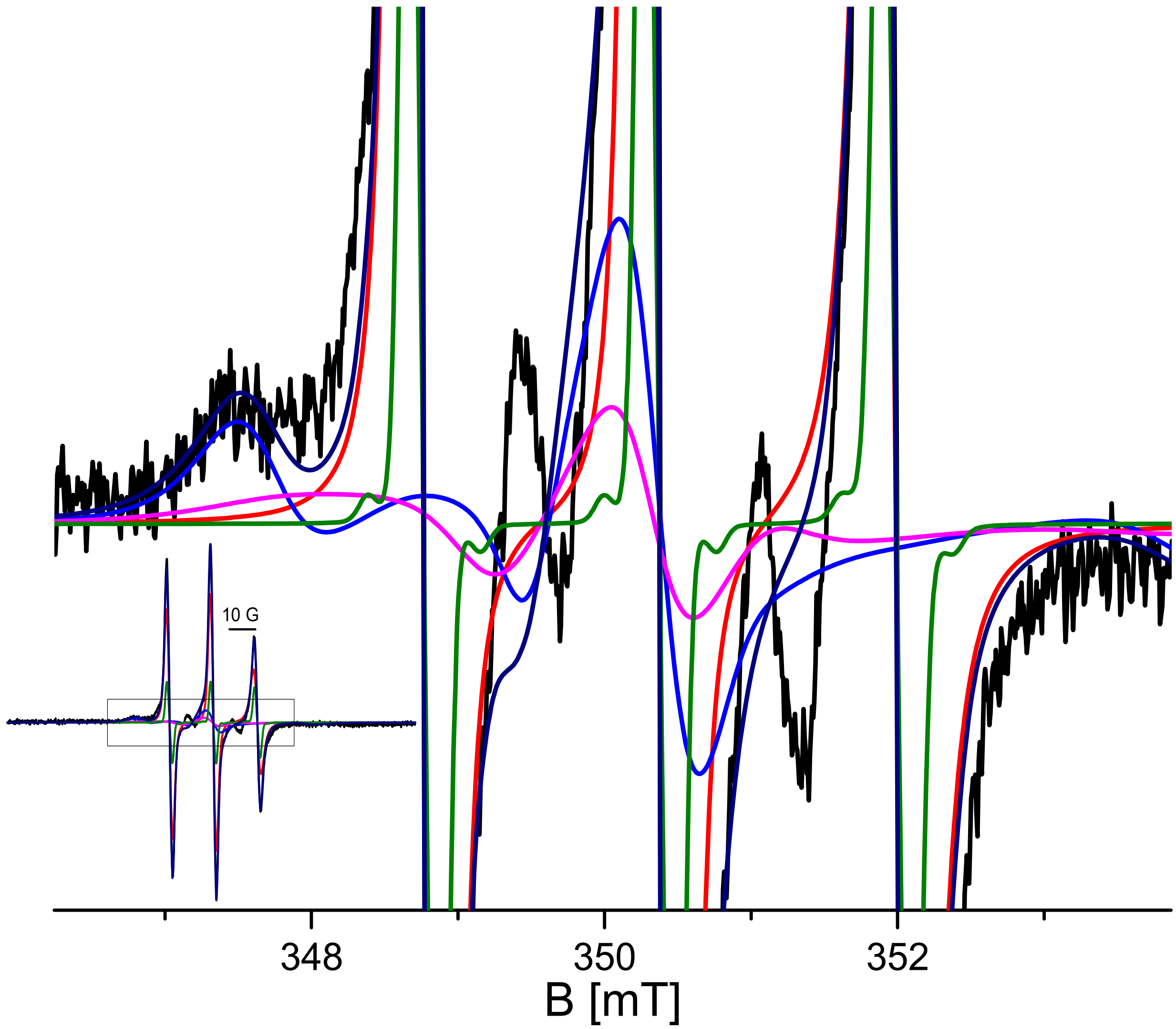

Figure S2 Spectral components used in the simulation of the EPR spectra of α-synuclein (18 hours of aggregation). Full spectra: Inset, box: zoomed in area. Zoomed-in spectra: Amplitude expanded ten-fold with respect to inset. Experimental spectra (black), fast component (red), medium component (pink), slow component (blue), free spin label (green). Total simulation (navy). For details see text.

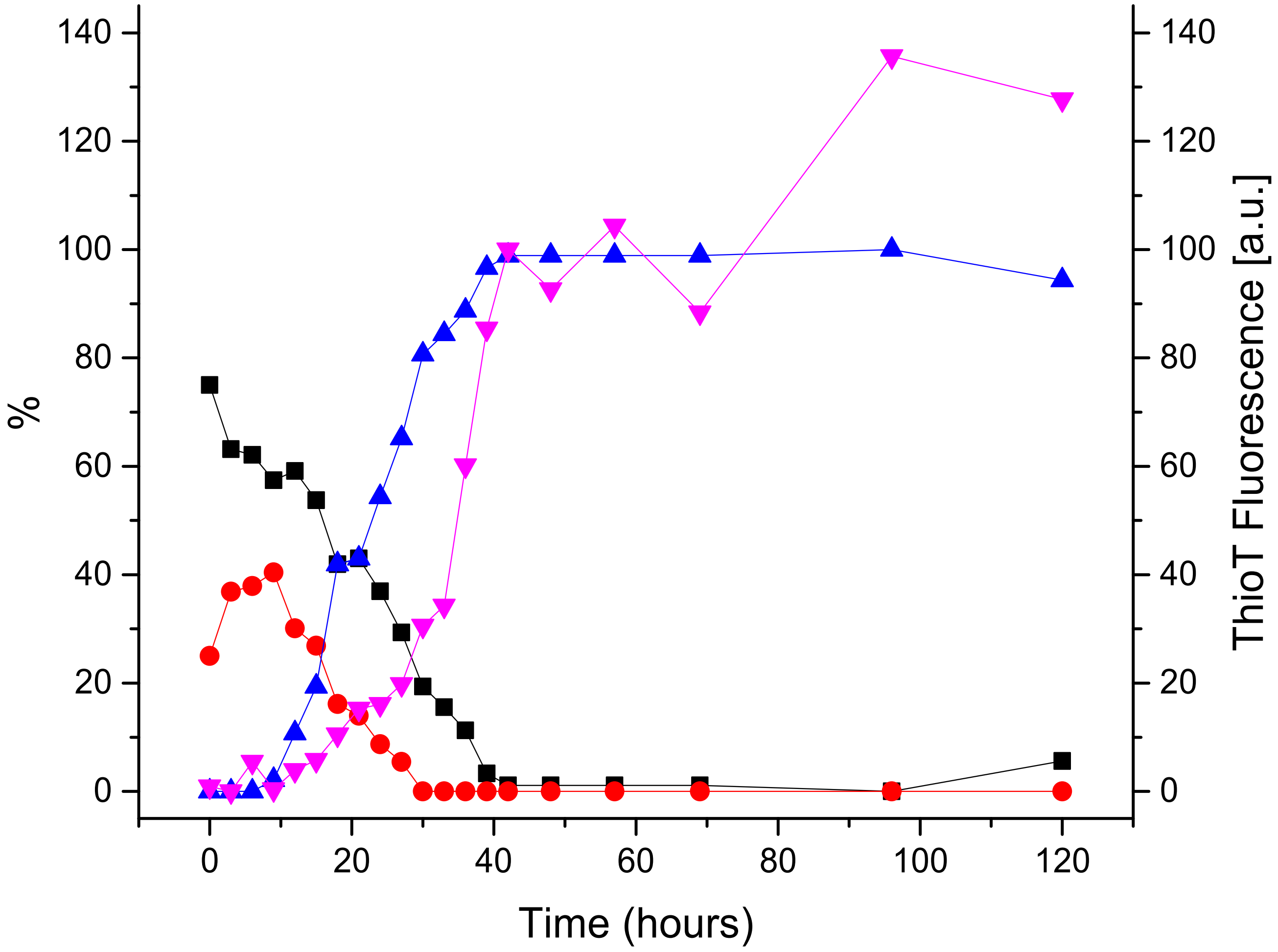

Figure S3 Aggregation of α-synuclein (R1-αS) as a function of time. Amount of fast fraction (black) caused by monomers. Amount of medium fraction (red) assigned to oligomers. Amount of slow fraction (blue) assigned to fibrils. Amount of fibrils derived from ThT fluorescence (pink, normalized to 100 at time 42 hours, the presumed plateau value). A 10 % component of free spin label was subtracted from the spectra from 3 to 120 hours. The lines are a guide to the eye.

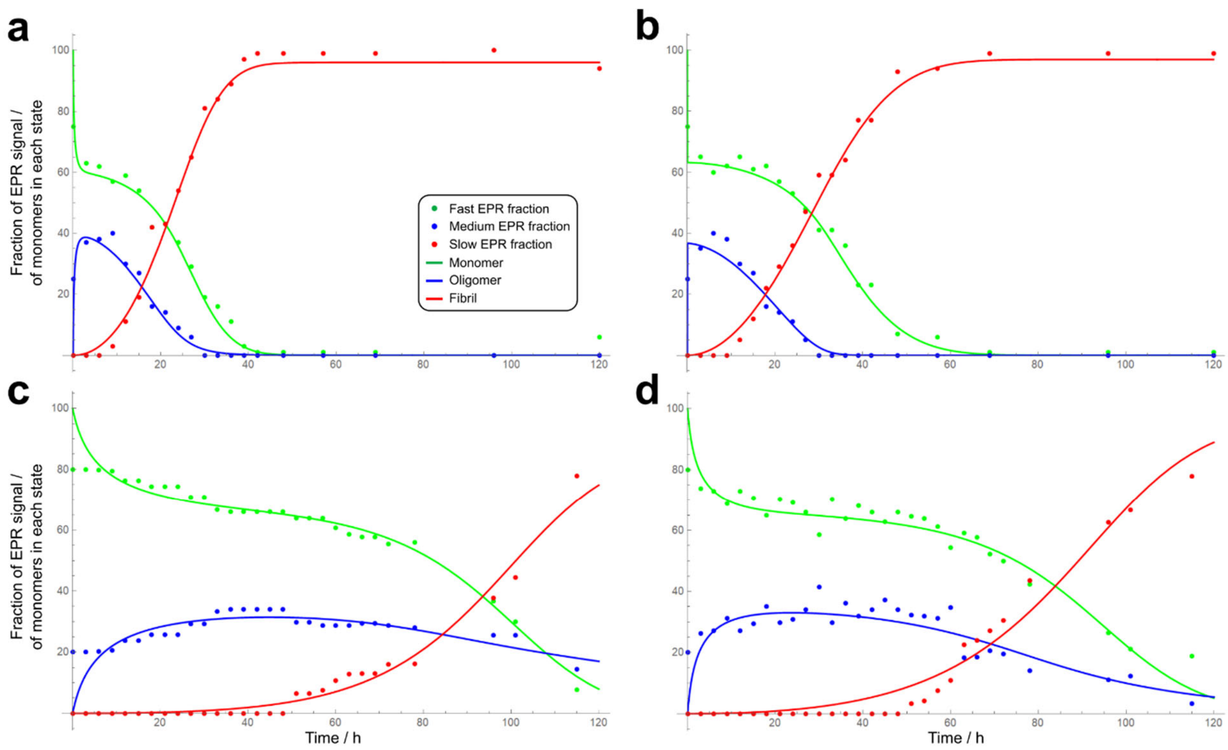

Figure S4 Additional αS-aggregation experiments showing reproducibility of results and effect of different surface to volume ratios and fits according to on-pathway model. Conditions: see main text. All aggregations performed in 2.0 ml LoBind Eppendorf tubes **a**: Data main text, aggregation volume 1.0 ml, sample LV1. **b**: Same conditions as **a**, sample LV2. **c:** sample with 1.5 ml aggregation volume, sample HV1 **d**: sample prepared as **c**, sample HV2. For detailed sample conditions, see text. Kinetic fits (solid lines) assuming an on path model with an oligomer reaction order of n=7.

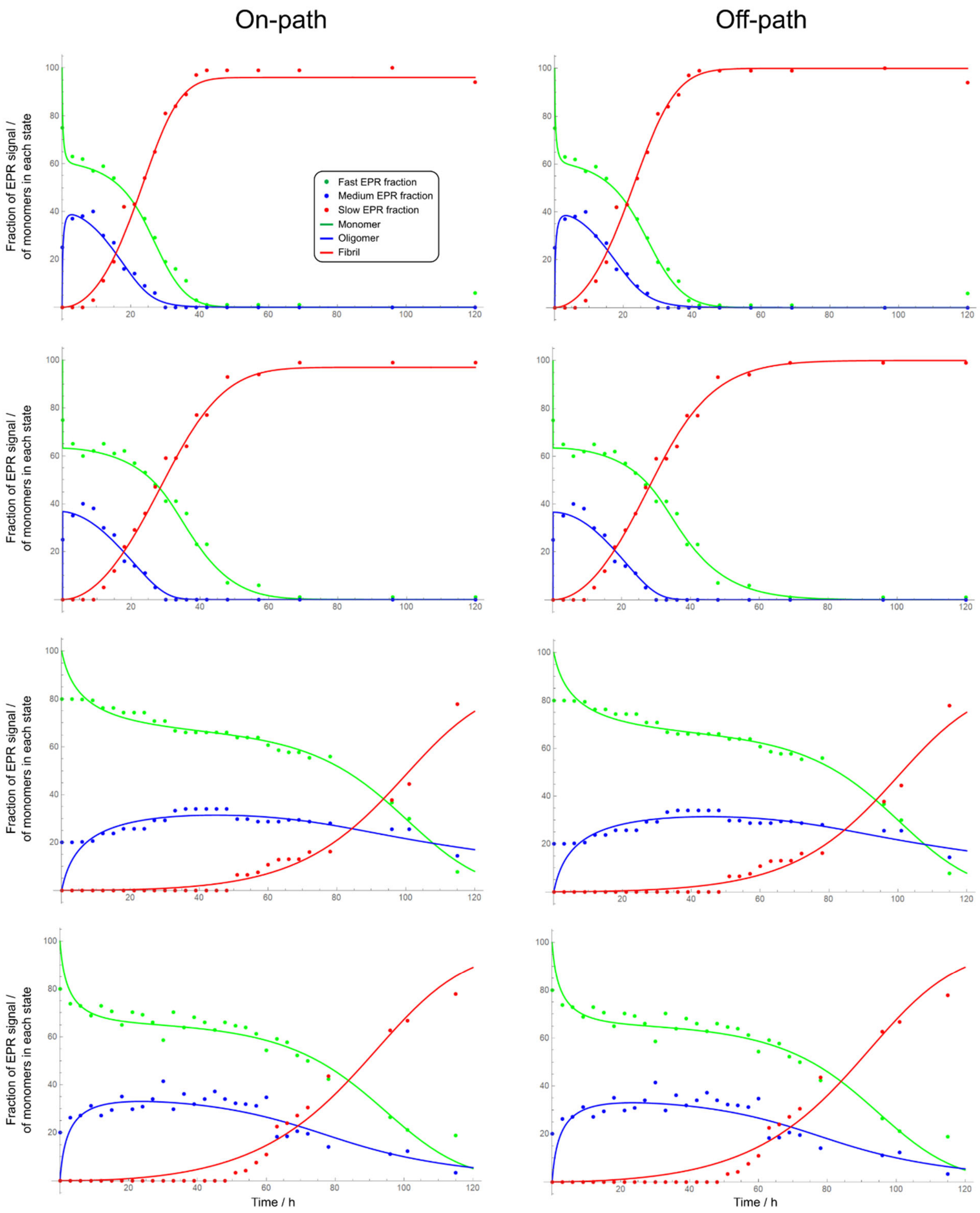

Figure S5 Comparison of on- and off-path fits: For all datasets, both the fits of an on-path model and the fits of an off-path model are consistent with the data.

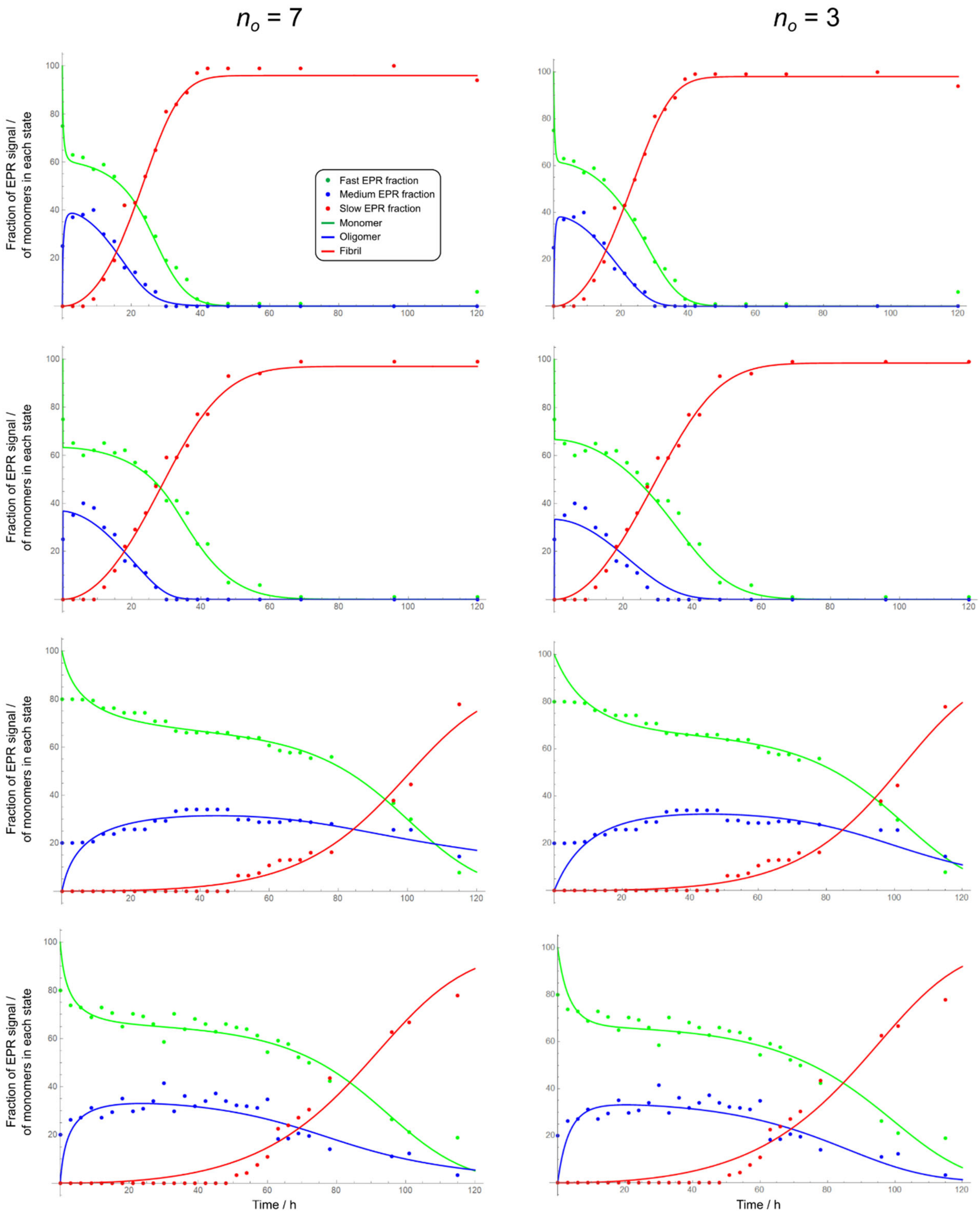

Figure S6 Fits of all datasets assuming oligomers are on-path and using an oligomer formation reaction order *n* = 7 (left column) or *n* = 3 (right column). While n=7 performs slightly better, the differences are not very pronounced.

References

1 Naiki H, Higuchi K, Hosokawa M, Takeda T. Fluorometric determination of amyloid fibrils in vitro using the fluorescent dye, thioflavine T. *Anal Biochem* 1989; **177**: 244–249.
